# Synthetic dye decolorization by three sources of fungal laccase

**DOI:** 10.1186/1735-2746-9-27

**Published:** 2012-12-15

**Authors:** Hamid Forootanfar, Atefeh Moezzi, Marzieh Aghaie-Khozani, Yasaman Mahmoudjanlou, Alieh Ameri, Farhad Niknejad, Mohammad Ali Faramarzi

**Affiliations:** 1 Department of Pharmaceutical Biotechnology, Faculty of Pharmacy and Biotechnology Research Center, Tehran University of Medical Sciences, P.O. Box 14155-6451, Tehran, 14174, Iran; 2 Herbal and Traditional Medicines Research Center, Kerman University of Medical Sciences, Kerman, Iran; 3 Department of Laboratory Sciences, Faculty of Health, Golestan University of Medical Sciences, Gorgan, Iran

**Keywords:** Decolorization, Removal, Hydroxybenzotriazole, Laccase, Synthetic dyes, Oxidase

## Abstract

Decolorization of six synthetic dyes using three sources of fungal laccase with the origin of *Aspergillus oryzae*, *Trametes versicolor*, and *Paraconiothyrium variabile* was investigated. Among them, the enzyme from *P. variabile* was the most efficient which decolorized bromophenol blue (100%), commassie brilliant blue (91%), panseu-S (56%), Rimazol brilliant blue R (RBBR; 47%), Congo red (18.5%), and methylene blue (21.3%) after 3 h incubation in presence of hydroxybenzotriazole (HBT; 5 mM) as the laccase mediator. It was also observed that decolorization efficiency of all dyes was enhanced by increasing of HBT concentration from 0.1 mM to 5 mM. Laccase from *A. oryzae* was able to remove 53% of methylene blue and 26% of RBBR after 30 min incubation in absence of HBT, but the enzyme could not efficiently decolorize other dyes even in presence of 5 mM of HBT. In the case of laccase from *T. versicolor*, only RBBR was decolorized (93%) in absence of HBT after 3 h incubation.

## Introduction

More than 10,000 various dyes stable to light, chemicals and microbial degradation are manufactured and used by textile, cosmetic, plastic and printing industries [1-3]. Based on the chemical structure of chromogenic groups, dyes are classified as azo, heterocyclic/polymeric or triphenylmethanes [4,5] and about 60% of produced dyes belong to the azo group which are categorized as monoazo, diazo, and triazo dyes [1].

Discharge of wastewater containing synthetic dyes especially polyaromatics and their carcinogenic health effects as an environmentally important problem, persuaded environmental engineers to develop new techniques for treatment of such hazardous compounds [6-9]. Beside conventional physicochemical methods [9], application of fungal and bacterial strains capable of adsorbing or degrading [1,9,10] of different dye groups has been considered as a novel concern in this field during last decades. Comparing to physicochemical methods viz., precipitation, filtration, adsorption, and TiO_2_ oxidation [11] the enzymatic treatment of dyes have low energy cost and is a more ecofriendly process not yet commonly used in the textile industries [5,12-14].

The copper containing oxidase, laccase (benzenediol oxygen oxidoreductase, EC 1.10.3.2), which is mainly produced by white-rot basidomycetes and other fungal [15] and bacterial strains [16] and also some plants [15] have been used in various biotechnological and environmental processes. Lack of substrate specificity introduced laccase as an enzyme able to oxidize wide range of chemical compounds such as diphenols, polyphenols, diamines, aromatic amines, benzenethiols, and substituted phenols [17-20] as well as different groups of colored pollutants [2,4]. In contrast to other oxidases such as peroxidases, laccase requires no H_2_O_2_ for oxidation reaction [15,20]. Such properties make laccase s an important enzyme in biodegradation of xenobiotics and phenolic compounds and decolorization of dyes [2,21].

Decolorization of a wide range of synthetic and textile dyes using laccases from the genus of *Trametes* (from basidomycete family) has been investigated in recent years [5,15]. For example, Maalej-Kammoun et al. [4] studied on malachite green decolorization ability of a newly isolated strain of *Trametes* sp. Furthermore, the laccase from genetically modified *Aspergillus oryzae* (DeniLite IIS) was applied for elimination of a large number of reactive textile dyes and other xenobiotics [22-24].

The aim of the present study was to evaluate decolorization ability of three sources of laccase obtained from *Paraconiothyrium variabile*, *Trametes versicolor* and *Aspergillus oryzae* on six synthetic dyes. The effect of hydroxybenzotriazole (HBT) as the laccase mediator on dye removal was also investigated.

## Methods

### Chemicals

2,2’-Azinobis-(3-ethylbenzthiazoline-6-sulphonate) (ABTS) was provided by Sigma-Aldrich (St. Louis, MO, USA). All of the dyes used in the present study (Table 1) were purchased from Merck Co. (Darmstadt, Germany). Commercial laccases including the pure enzyme of *T. versicolor* (20 U/mg) and laccase with the origin of *A. oryzae* (Denilite IIS; 120 LAMU/g) were supplied by Sigma-Aldrich (St. Louis, MO, USA) and Novozyme (Novozymes A/S, Denmark), respectively. All other chemicals were of analy-tical grade.

**Table 1:** Names, classification and maximum absorbance (in the citrate buffer 0.1 M, pH = 4.5) of six dyes used

**Name**	**Classification**	**Dye concentration (mg/L)**	**λ**_**max**_
Panseu-S	Diazo	240	513
Methylene blue	Heterocyclic	100	610
Congo red	Diazo	360	514
Bromophenol blue	Triphenylmethane	120	592
Commassie brilliant blue	Triphenylmethane	120	583
Remazol Brilliant Blue R (RBBR)	Anthraquinone	600	592

^a^[25].

### Optimization of laccase production by P. variabile

Besides the two above-mentioned commercial laccases, the culture broth from optimized medium (with laccase activity of 16678 U/L) of a laccase producing ascomycete, *P. variabile*[26], which was previously investigated [27] was also applied for decolorization studies.

### Determination of laccase activity

Laccase activity was determined using ABTS as the substrate [28,29]. The reaction mixture consisted of 0.5 mL ABTS (5 mM) dissolved in 100 mM acetate buffer (pH = 4.5) and 0.5 mL of enzyme solution or culture supernatant (at desired dilution) followed by incubation at 37°C and 120 rpm. Oxidation of ABTS was monitored by an increase in absorbance at 420 nm (ε_420_ = 36,000/M cm) [30]. One unit of laccase activity was defined as the amount of enzyme required to oxidize 1 μmol of ABTS/min.

### Decolorization experiments

To study on decolorization ability of three mentioned laccase sources, 0.5 mL of laccase solution (in the case of laccases from *A. oryzae* and *T. versicolor*, enzyme powders were dissolved in citrate buffer 0.1 M pH = 4.5 to reach the activity of 16.7 U/mL and in the case of *P. variabile* 0.5 mL of the optimized culture broth) was added to 2 mL of each dye solution followed by incubation in a rotary shaker (35°C and 120 rpm) for 3 h. Samples of 1 mL volume were taken from each reaction mixture and decrease in the maximum absorbance was recorded every 30 min. The concentration and maximum absorbance of each dye are summarized in Table 1. Percent of dye decolorization was calculated as the formula: decolorization (%) = [(Ai-At)/Ai] × 100, where, Ai: initial absorbance of the dye, At: absorbance of the dye at any time interval [23]. Negative controls (reaction mixtures without enzyme) were designed as a reference to compare decolorization percent of treated samples. Each decolorization experiment was performed in triplicate and mean of decolorization percents were reported.

### Effect of HBT concentration on the decolorization

In order to study of the effect of HBT as the laccase mediator on decolorization, same experiments (as mentioned above) were done by incorporation of HBT in reaction mixture to reach final concentrations of 0.1 mM, 1 mM and 5 mM.

## Results

### Bromophenol blue removal in presence of three laccase

**Figure 1:**
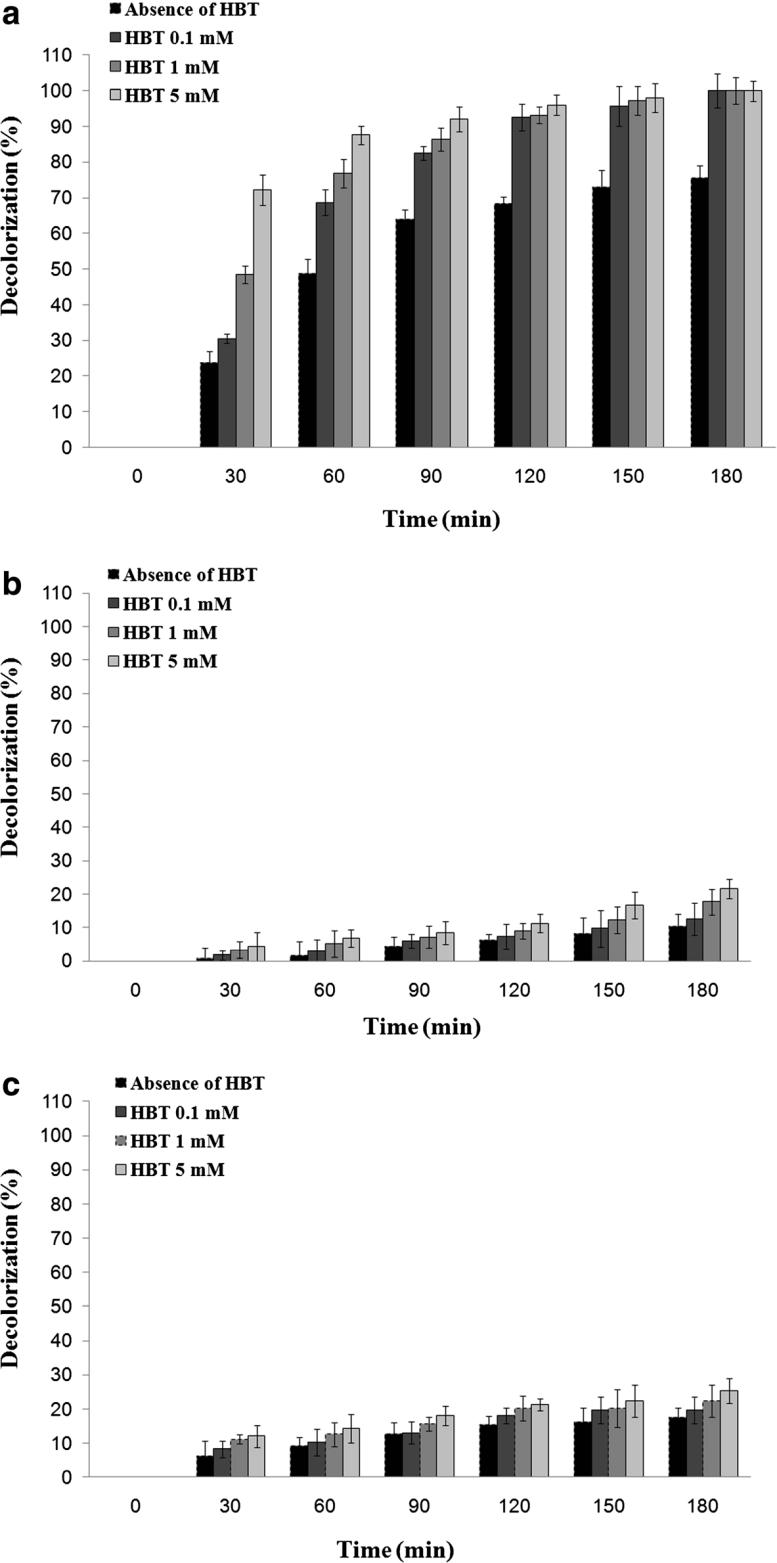
**Profile of decolorization of bromophenol blue (initial concentration of 120 mg/L) in absence and presence of HBT using (a) optimized culture broth of *P. variabile *(b) the laccase from *A. oryzae *and (c) purified laccase of *T. versicolor*.**

As shown in Figure 1, the laccase of *P. variabile* was the most efficient enzyme with 72.2% removal of bromophenol blue (a triphenylmethane dye) after 30 min treatment in absence of HBT. However, decolorization percent by using two other sources of fungal laccases did not increase higher than 25.3% even after 3 hours incubation (Figure 1).

### Commassie brilliant blue elimination using the applied laccases

**Figure 2:**
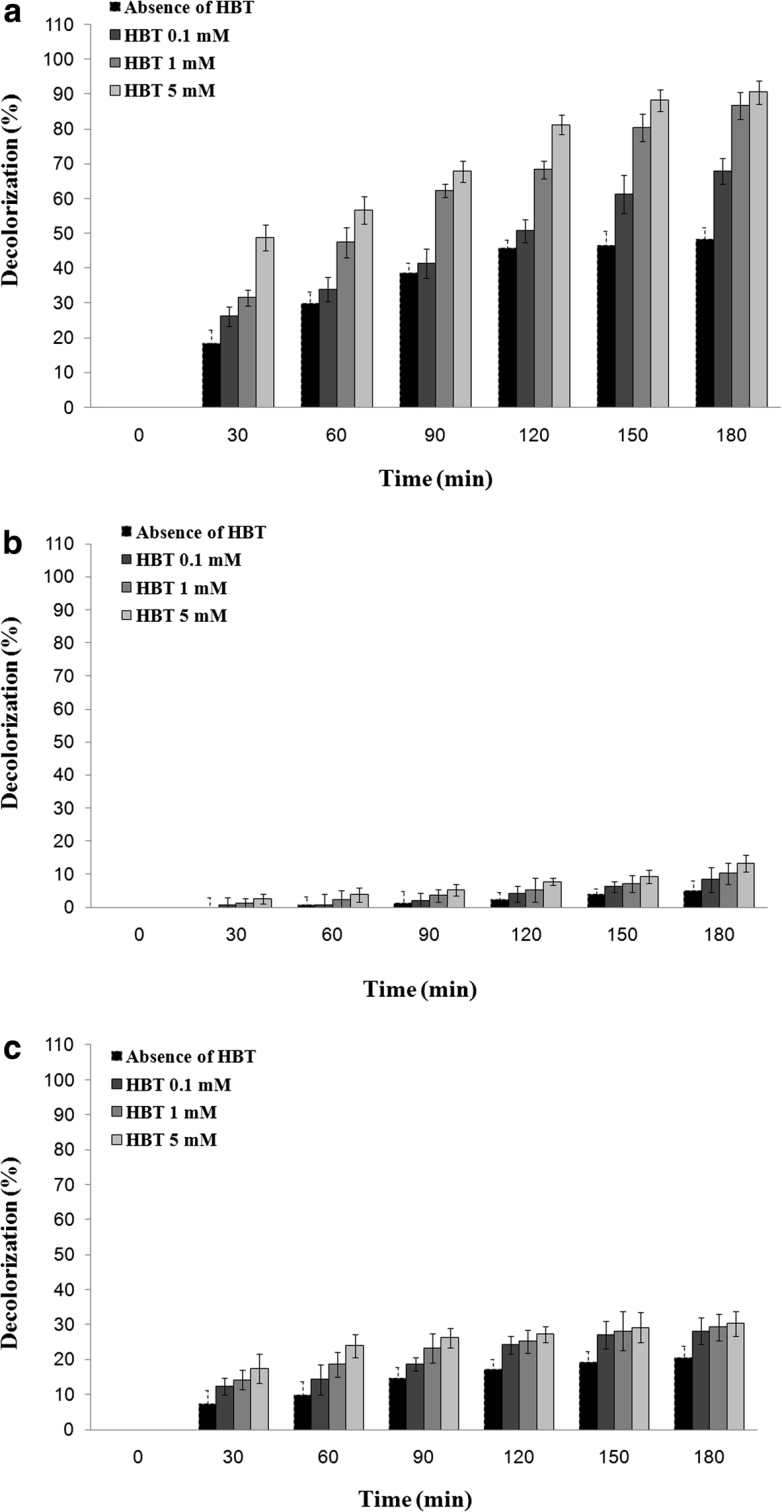
**Decolorization of commassie brilliant blue (initial concentration of 120 mg/L) assisted by (a) optimized culture broth of *P. variabile *(b) laccase of *A. oryzae *and (c) laccase from *T. versicolor *in absence and presence of laccase mediator HBT.**

Compare to laccases from *T. versicolor* and *A. oryzae* which represented 30.3% and 13.3% decolorization of commassie brilliant blue, respectively (Figure 2), the optimized culture broth of *P. variabile* could eliminate 90.6% of this triphenylmethane dye after 3 hours incubation in presence of HBT (5 mM).

### RBBR removal using three sources of laccase

**Figure 3:**
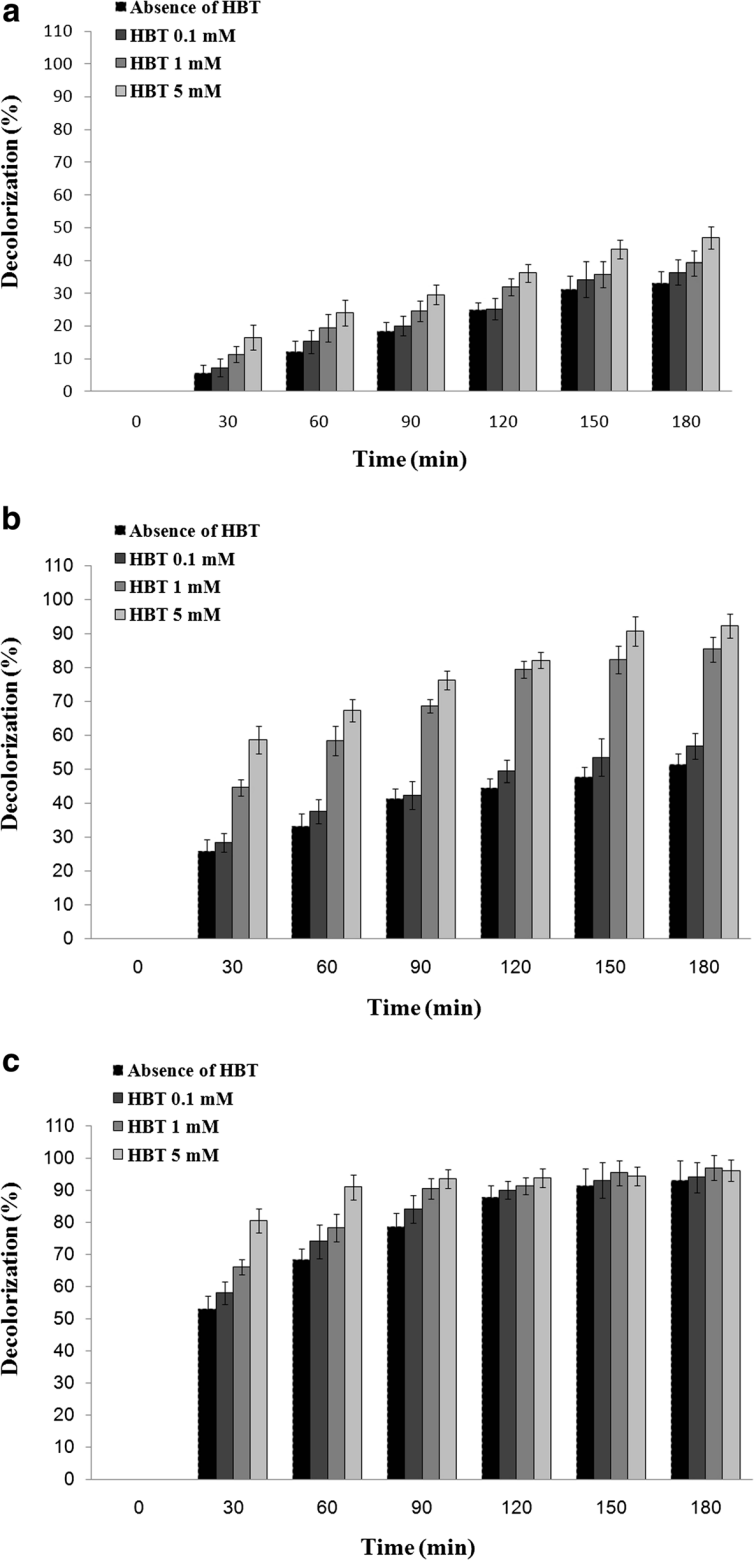
**Effect of (a) laccase of *P. variabile *(b) *A. oryzae *laccase and (c) purified laccase of *T. versicolor *on RBBR (initial concentration of 600 mg/L) in absence and presence of HBT.**

The influences of extracellular laccase from optimized culture broth of *P. variabile*, the laccase from *T. versicolor* and also the laccase of *A. oryzae* on the antraquinone dye of RBBR are presented in Figure 3. In all cases, decolorization percent increased by increasing of HBT concentration. The purified laccase of *T. versicolor* showed highest decolorization percent by 80.5% removal after 30 min incubation in absence of HBT. In the case of laccase from *A. oryzae* and in absence of HBT, decolorization percent was found to be 28.3% after 30 min. However, *P. variabile* represented only 16.6% decolorization at the same time in presence of HBT (5 mM).

### Decolorization of methylene blue assisted by different fungal laccases

**Figure 4:**
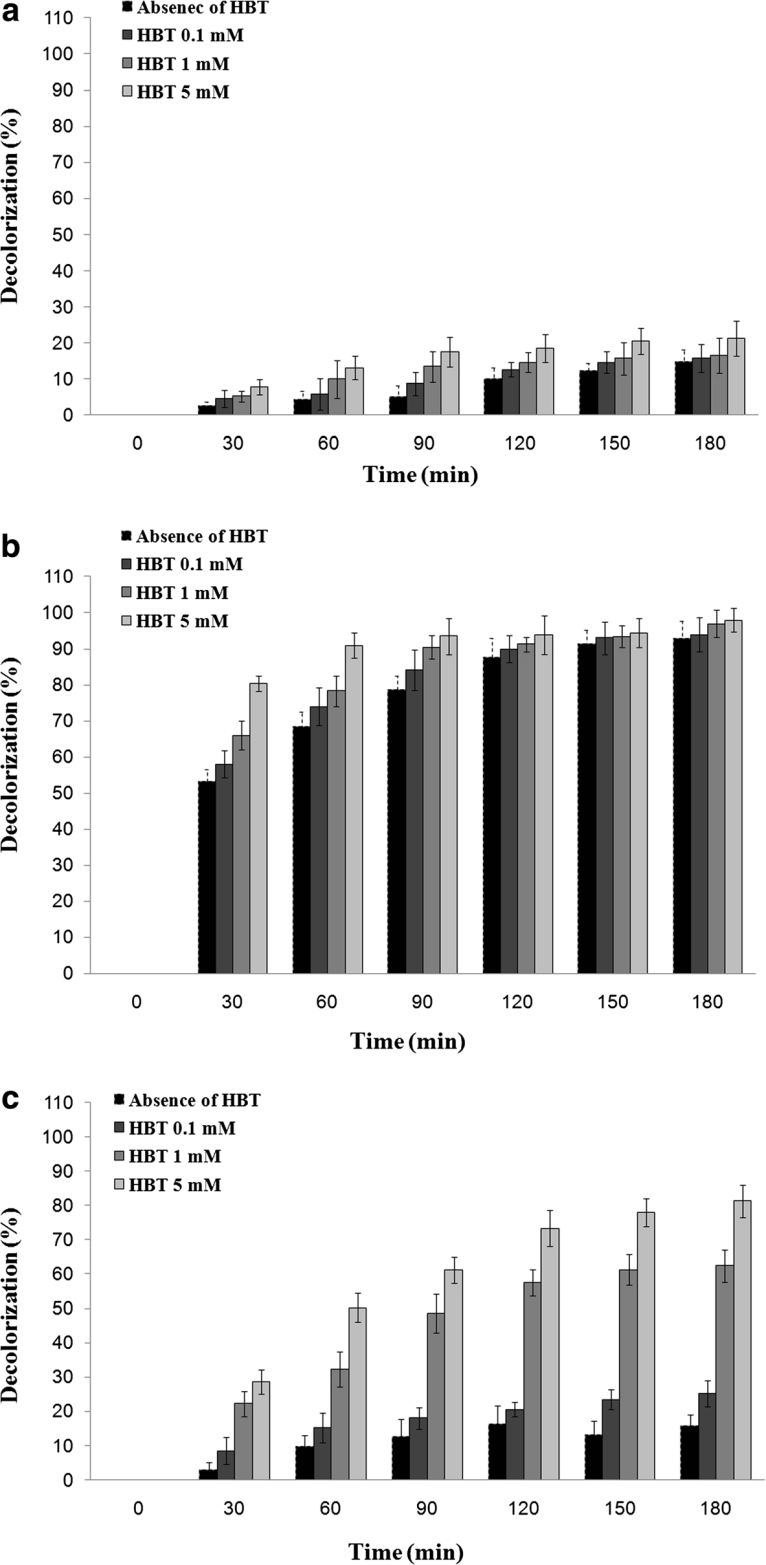
**Removal of methylene blue by (a) laccase of *P. variabile *(b) *A. oryzae *laccase and (c) purified laccase of *T. versicolor *in presence and absence of HBT.**

As shown in Figure 4, the laccase of *P. variabile* could not efficiently eliminate the methylene blue (a heterocyclic dye) and after 3 hours treatment in presence of mediator (HBT 5 mM) only 21.3% decolorization was achieved. However, two other sources of laccase showed 98% (laccase of *A. oryzae*) and 81.3% (laccase from *T. versicolor*), respectively, decolorization through this synthetic dye.

### Decolorization of panseu-S and Congo red using three fungal laccases

**Figure 5:**
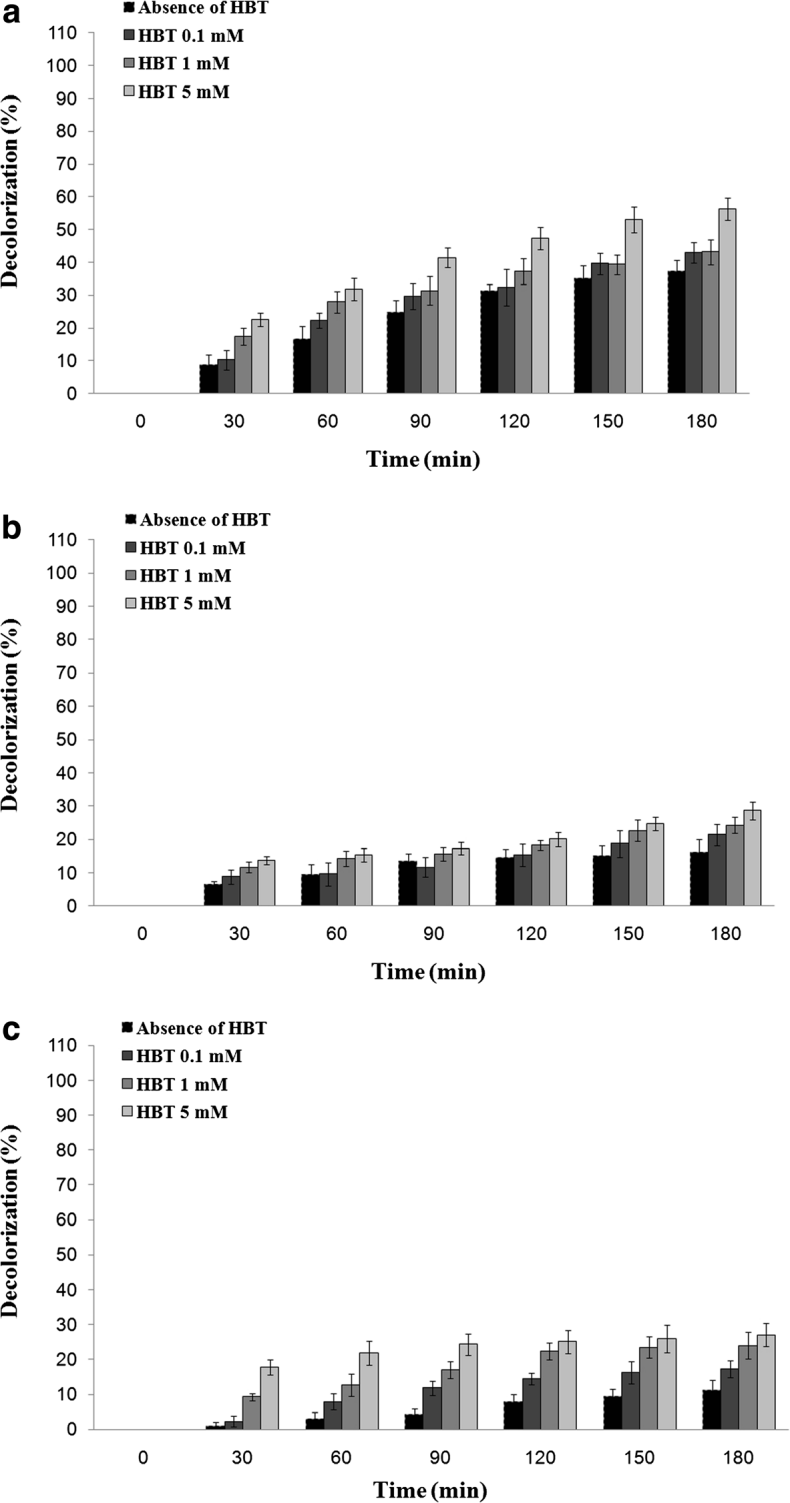
**Treatment of 240 mg/L of panseu-S using: (a) optimized culture broth of *P. variabile *(b) the laccase from *A. oryzae *and (c) purified laccase of *T. versicolor *in presence of different concentration of HBT as laccase mediator.**

**Figure 6:**
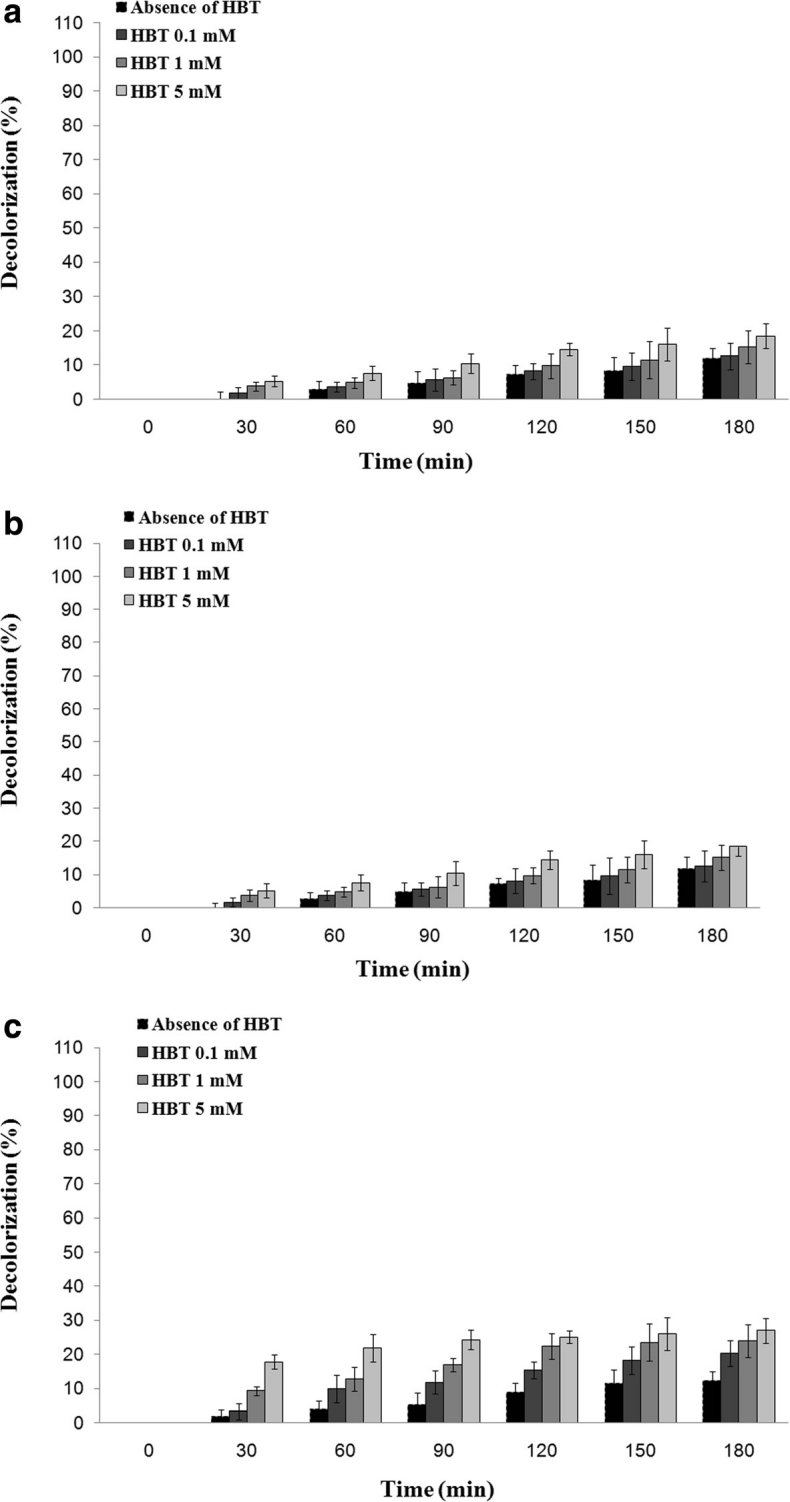
**Decolorization curves of Congo red (initial concentration of 360 mg/L) in presence of (a) optimized culture broth of *P. variabile *(b) the laccase from *A. oryzae *and (c) purified laccase of *T. versicolor *and different concentration of HBT.**

These two diazo dyes were the most resistant dyes through enzymatic treatment (Figures 5 and 6). In the case of panseu-S, decolorization percent of both of the laccases from *A. oryzae* and *T. versicolor* did not reach higher than 28% even in presence of highest concentration of laccase mediator (5 mM) (Figure 5). However, in the same condition the optimized culture broth of *P. variabile* showed dye removal percent of 56.3% (Figure 5). All of the three mentioned laccases showed lowest decolorization percent in the case of Congo red (Figure 6).

## Discussion

Laccase producing microorganisms especially white rot fungi were extensively applied for dyes decolorization experiments. Decolorization ability of five indigenous white rot fungi on vat dyes during 10 days was studied by Asgher et al. [31] and it was determined that *Coriolus versicolor* IBL-04 showed excellent decolorization potential on all tested dyes. Decolorization potential of laccases even on a same dye shows variation and depends on the biological sources of producing microorganism. For example, 60.5% of malachite green (with initial concentration of 60 mg/L) was removed after 15 min incubation of the dye in presence of laccase from *P. variabile*[26] while Zhou et al. [32] reported 98% of malachite green decolorization using laccase of *Ganoderma* sp. En3 after 72 h incubation. In the present study, the pure laccase of *T. versicolor* (Syn. *Coriolus versicolor*) could not efficiently decolorize the tested synthetic dyes except for RBBR and methylene blue during 3 h of incubation. The optimized culture broth of *P. variabile* showed excellent decolorization potential while the laccase with the origin of *A. oryzae* was able to decolorize methylene blue (a heterocyclic dye) and RBBR (an antraquinone dye). Desouza et al. [2] investigated decolorization capacity of the laccase from a fungal isolated strain (designed as NIOCC # 2a) on nine synthetic dyes and revealed that such laccase decolorized RBBR (46%), methylene blue (5%) and Congo red (47%) after 12 h incubation and production of the laccase was increased in presence of the tested dyes.

Comparing to other dye groups, triphenylmethane dyes are resistant to enzymatic treatment and need longer time for decolorization [12]. However, in a recent study, it was showed that the laccase of *P. variabile* decolorized 60.5% of malachite green (with initial concentration of 60 mg/L) after 15 min incubation [26]. The present work revealed that optimized culture broth of the laccase producing ascomycete was able to remove two other triphenylmethane dyes (bromophenol blue and commassie brilliant blue) efficiently. In the study of Zhou et al. [32], 98.3% of bromophenol blue (with initial concentration of 50 mg/L) was decolorized after 72 h incubation. Generally, anthraquinone dyes are suitable substrate for laccase [33]. Three laccase sources applied in the present work was efficiently removed RBBR. Similar results were reported by Zeng et al. [33] indicated 87% and 77% decolorization of RBBR and reactive blue 4 (two typical anthraquinone dyes), respectively, by laccase from *Trametes trogii* SYBC-LZ.

HBT is a synthetic laccase mediator assisting in laccase oxidation of different substrates by facilitating of electron transfer from O_2_ to laccase substrate [15]. In the present work, decolorization percentages of all studied dyes were found to enhance in presence of HBT as a laccase mediator. Same results were reported in the study of Maalej-Kammoun et al. [4] where they found that HBT showed the highest decolorization of malachite green among ten laccase investigated laccase mediators.

## Conclusion

In conclusion, three sources of fungal laccase were applied for decolorization of six synthetic dyes among which the laccase with the origin of *P. variabile* was able to remove all tested dyes. The laccase from *A. oryzae* was not able to decolorize examined dyes except for methylene blue and RBBR. In absence of HBT, RBBR was the sole synthetic dye efficiently removed by laccase from *T. versicolor*.

## Competing interests

The authors declare that they have no competing interests.

## Authors’ contributions

HF assisted in the writing of the manuscript and analyzing of data. AM carried out decolorization studies. Production of laccase using optimized culture broth of *P. variabile* was performed by MA-K. YM participated in decolorization studies. AA participated in reviewing of the manuscript and decolorization studies. FN contributed in writing of the manuscript and decolorization studies. MAF involved in purchasing of required materials and instruments, designing of decolorization experiments, analyzing of data and reviewing of the manuscript. All authors read and approved the final manuscript.
